# Hybrid Algorithms for Fuzzy Reverse Supply Chain Network Design

**DOI:** 10.1155/2014/497109

**Published:** 2014-04-24

**Authors:** Z. H. Che, Tzu-An Chiang, Y. C. Kuo, Zhihua Cui

**Affiliations:** ^1^Department of Industrial Engineering and Management, National Taipei University of Technology, Taipei 10608, Taiwan; ^2^Department of Business Administration, National Taipei College of Business, Taipei 10051, Taiwan; ^3^Complex System and Computational Intelligent Laboratory, Taiyuan University of Science and Technology, Taiyuan 030024, China; ^4^State Key Laboratory for Novel Software Technology, Nanjing University, Nanjing 210023, China

## Abstract

In consideration of capacity constraints, fuzzy defect ratio, and fuzzy transport loss ratio, this paper attempted to establish an optimized decision model for production planning and distribution of a multiphase, multiproduct reverse supply chain, which addresses defects returned to original manufacturers, and in addition, develops hybrid algorithms such as Particle Swarm Optimization-Genetic Algorithm (PSO-GA), Genetic Algorithm-Simulated Annealing (GA-SA), and Particle Swarm Optimization-Simulated Annealing (PSO-SA) for solving the optimized model. During a case study of a multi-phase, multi-product reverse supply chain network, this paper explained the suitability of the optimized decision model and the applicability of the algorithms. Finally, the hybrid algorithms showed excellent solving capability when compared with original GA and PSO methods.

## 1. Introduction

Many scholars have been devoted to the study of positive supply chains, for instance [1–5], however, the reverse supply chain has seldom been involved. Dowlatshahi [6] pointed out that reserve logistics, which is a new concept in logistics and supply chain management, offer efficient reverse logistics management that could improve the competitive power of the enterprises, especially when they face fierce competition with low-margin profits. Notwithstanding price and quality as important, influential marketing factors within a complex product path, business operations should focus on how to offer improved after-sale services to customer. Setting its target on household appliances and computers in 3C (Computers, Communications, Consumer Electronics).

Shih [7] discussed the reverse logistics system for end-of-life of products, viewing reverse logistics systems as a crucial element of future business operations; more efforts should be made in reutilization, rework, and recycling at end-of-life [8]; reverse logistics is a process of recycling and refabricating materials [9]; reverse logistics is helpful to protect environments and reduce waste of resources, thus providing an opportunity for the reutilization of products. Ko and Evans [10] proposed a positive and a reverse supply chain network, based on a third party's logistics, and constructed a mixed-integer nonlinear planning model of a dynamic integrated distribution network, taking into account a two-echelon supply chain network and capacity constraints, underlining the significance of returning the defective products to the suppliers' partners. In addition, the defect ratios and transport loss ratios are fuzzy values in the real-world supply chain environments. Fuzzy defect ratios and fuzzy transport loss ratios, thus, are included in this reverse model for meeting actual conditions.

Thus, this paper focuses on the reverse supply chain and constructs an optimized decision model for the selection of supply chain partners as well as determination of production and distribution quantities. Multiechelon logistics issues could be regarded as a Knapsack of a multiple selection portfolio, and resource distribution as a NP-hard issue [11]; however, the reverse supply chain is more complex in this paper, with production defect ratios, transport loss ratios, and designated reprocessing are considered.

GAs have been widely used in solving real-world complex optimization problems [12–16] and Min et al. [17], Sha and Che [18], Tsai [19], Ko and Evans [10], Min et al. [20], Che and Chiang [21], and Che and Chiang [22] applied GA to solve the planning issues of a supply chain network. Moreover, PSOs also have been widely employed for solving optimization problems in different fields and some heuristic algorithms with similar concept of PSO are developed [23–30]. Yin and Wang [31], Yu and Fang [32], Chen et al. [33], Che and Cui [34], and Che [35] employed a PSO to optimize a resource distribution system with satisfactory results. Yet, no research efforts were made with respect to the planning of a reverse supply chain with GA and PSO. For this reason, these two heuristic algorithms were used to solve the reverse supply chain in this paper, along with other three hybrid algorithms: PSO-GA, PSO-SA, and GA-SA. The solving capability of these five algorithms was compared, and the optimum one was selected as a reference for decision-making.

## 2. Fuzzy Theory

Fuzzy theory, first proposed by Zaden in 1965 as an extension of a general set, is a numerical control methodology for imitating human thought and addressing the inaccuracy of all physical systems. According to Fuzzy Theory, the thought logic of human begins as fuzzy and is intended for judgment, even if conditions and data are uncertain, while modern computers feature bipolar logic, that is 0 or 1, different from the logic of humans. However, fuzzy logic theory can represent the degree of fuzzy concepts with values between 0 and 1, namely, “membership function.”

Karkowski [36] indicated that triangular is the most common membership function in solving possibilistic mathematical programming problems among the various types of membership function. Other related studies concerning the use of triangular fuzzy numbers for decision making problems are [37–40]. Hence, in this paper, the defect ratios and transport loss ratios are denoted by triangular fuzzy numbers. The triangular fuzzy numbers are not compulsory, if other types of fuzzy numbers are more applicable, they can be used.

**Figure 1:**
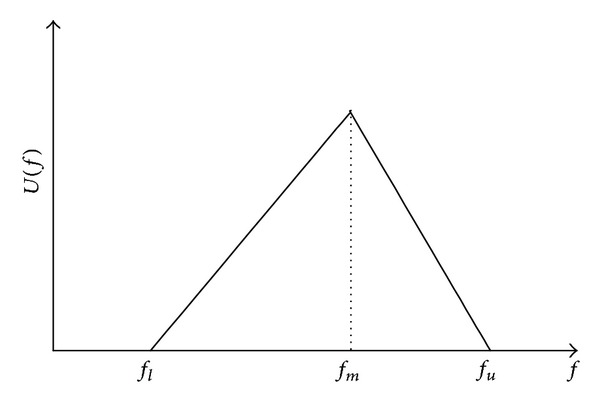
Membership functions of a triangular fuzzy number.

The triangular fuzzy number can be represented as *f* = (*f*_*l*_, *f*_*m*_, *f*_*u*_), where *f*_*l*_, *f*_*m*_, and *f*_*u*_ are lower bound, mode, and upper bound values, respectively.  Figure 1 shows the triangular possibility distribution of fuzzy number *f*.

In addition, it is essential for practical applications that a fuzzy number should be transformed to a numerical value. The transform process is called “defuzzification.” Associated ordinary number (AON) is a simple method in defuzzification and many researches have employed it directly and effectively [41–43]. In this paper, hence, the AON is used to find the crisp value for defect and transport loss ratios. The AON method can be expressed as

(1) $$\begin{array}{c} \text{AON}\left(f\right)=\frac{f_{l}+2f_{m}+f_{u}}{4}. \end{array}$$

## 3. Problem Formulation and Solving

### 3.1. Description and Definition

This paper analyzed the reverse distribution activities of defective products in a complex supply chain network, and explored a method of feeding these products back to the manufacturing partners, with consideration of the capacity constraints of suppliers, and the demands of multi-phase, multi-product production and planning. Moreover, the partners shall be selected based on total objective functions (minimized production, transport, inspection, rework costs, and optimized production quality), in addition, the fuzzified production defect ratio and transport loss ratio are taken into account. Given the fact of numerous suppliers in a supply chain network, with different production characteristics and resource allocation capabilities covering their capacity and yield, it is crucial to select proper suppliers in a complex supply chain network.

Positive logistics are highlighted in a traditional supply chain, but unavoidable defects arising from production or transportation processes are overlooked. In practice, the defective products must be returned to the manufacturers. Relevant original data in this paper were subjected to *T*-treatment for data integration [44–46]. Equation (2) is a *T*-treatment formula (*X*: variable, $\bar{X}$: average, SD: standard deviation).

(2) $$\begin{array}{c} T=\frac{X-\bar{X}}{\text{SD}/10}+50. \end{array}$$

### 3.2. Mathematical Model for Multiphase and Multiproduct Reverse Supply Chain

The mathematical symbols of a reverse supply chain one listed in the Symbols of Mathematical Model section.

The mathematical model of a multi-phase plan of a reverse supply chain is detailed as follows:

(3) Minimize [∑p=1P ∑i=1I−1 ∑j=1Ji ∑k=1Ki+1(PrC(i,j)×X((i,j),(i+1,k))p)      +∑p=1P ∑i=1I ∑j=1Ji ∑k=1Ki+1(TrC((i,j),(i+1,k))              ×(X((i,j),(i+1,k))p             +RX((i+1,k),(i,j))p))      +∑p=1P ∑i=II ∑j=1Ji ∑k=1Ki−1(PrC(i,j)×X((i−1,k),(i,j))p)      +∑p=1P ∑i=1I ∑j=1Ji ∑k=1Ki+1(ChC(i,j)×X((i,j),(i+1,k))p)]     ×∑p=1P ∑i=1I ∑j=1JiQ(i,j)p,

(4) s.t ∑k=1Ki+1X((i,j),(i+1,k))p=INT{∑k=1Ki+1X((i,j),(i+1,k))p    ×(1−TRF((i,j),(i+1,k)))+RX((i+1,k),(i,j))p−1},   i=1; p=1,2,3,…,P; j=1,2,3,…,J

(5) ∑k=1Ki−1X((i−1,k),(i,j))p=INT{[∑k=1Ki−1X((i−1,k),(i,j))p×∑k=1Ki+1(1−TRF((i,j),(i+1,k)))]    +∑k=1Ki+1RX((i+1,k),(i,j))p−1−∑k=1Ki−1RX((i,j),(i−1,k))p},i=2,…,I−1; p=1,2,3,…,P; j=1,2,3,…,J,

(6) ∑k=1Ki−1X((i−1,k),(i,j))p=INT{∑k=1Ki−1[X((i−1,k),(i,j))p    ×(1−TRF((i,j),(i−1,k)))       −RX((i,j),(i−1,k))p]},i=I; p=1,2,3,…,P; j=1,2,3,…,J,

(7) ∑k=1Ki−1RX((i,j),(i−1,k))p=INT{(∑k=1Ki−1X((i−1,k),(i,j))p    +∑k=1Ki+1RX((i+1,k),(i,j))p−1)×DRF(i,j)},i=2,3,…,I; p=2,3,…,P; j=1,2,3,…,J,

(8) X(i,j) =INT{∑k=1Ki+1X((i,j),(i+1,k))p×(1−DRF(i,j))    ×(1−TRF((i,j),(i+1,k)))},i=1; p=1,2,3,…,P; j=1,2,3,…,J,

(9) X(i,j)=INT{∑k=1Ki−1X((i−1,k),(i,j))p×(1−DRF(i,j))    ×(1−TRF((i,j),(i+1,k)))},i=2,3,…,I−1; p=1,2,3,…,P;         j=1,2,3,…,J,

(10) Min⁡CAP(i,j)≤∑k=1Ki+1X((i,j),(i+1,k))p≤Max⁡CAP(i,j),i=1,2,…,I−1; p=1,2,3,…,P; j=1,2,3,…,J,

(11) X((i,j),(i+1,k))p>0 and∈integer  ∀i,j,k,p,

(12) RX((i,j),(i−1,k))p=0for  p=0;  i=1,2,…,I;j=1,2,3,…,J;  k=1,2,3,…,K.

Equation (3) shows objective functions involved in minimizing production costs, transport costs, rework costs, check costs, and quality (quality level 1 is defined as optimum quality, and 10 as worst quality); (4), (5), and (6) represent quantity transported from the first-hierarchy partner to the second-hierarchy partner, and finally to the partner of last hierarchy, respectively, while the fuzzy transport loss ratio and rework quantity of returned defective products are also considered; (7) represents the rework quantity of defective products to be returned to the partner of previous hierarchy; (8) and (9) indicate that limited production quantities of partners from first to last hierarchy shall meet customer demands; (10) indicates that the limited transport quantity will not be bigger than the maximum capacity, nor less than the minimum capacity of the partner; (11) indicates that the limited transport quantity must be an integer bigger than zero; and (12) indicates that no defective product is produced in the early phase of multi-product, multi-phase production planning.

### 3.3. Proposed Algorithms

In this paper, GA, PSO, PSO-GA, GA-SA, and PSO-SA were applied to determine the best approximate solution, with minimum objective functions, in a reverse supply chain.

#### 3.3.1. GA Solving Model

**Figure 2:**
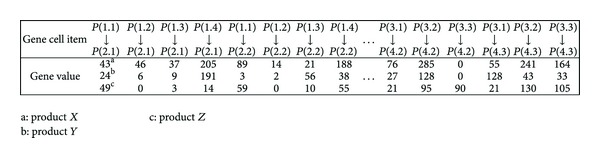
Structure of chromosome.

*Step  1*. Develop the structure of chromosome (Figure 2) and the production and transport quantities were real-coded. Eiben et al. [47] argued that real-coding could accelerate the algorithm convergence and improve consistency. In Figure 2, *P*(1.1) → *P*(2.1) represents the transport path from the first partner of the first hierarchy to the first partner of the second hierarchy, Gene value is an integral number representing the quantity of partner product transported on the distribution path.

*Step  2*. Generate a random initial population according to the final customer demands of the partners, restrict the population for meeting the demand constraint (4)~(12), and then obtain the Fitness value by substituting the chromosome into the objective formula (3). 

*Step  3*. Use Roulette Wheel method to select the chromosomes. Calculate selection probability of every chromosome, of which the chromosomes of better fitness function have a higher probability. 

**Figure 3:**
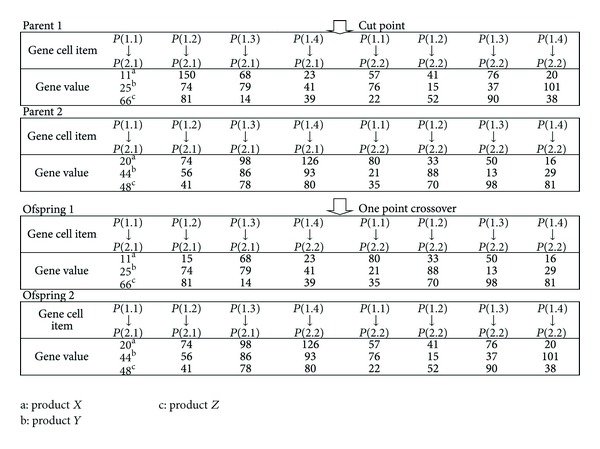
Single-point crossover.

*Step  4*. Randomly select two chromosomes from the population for crossover, and generate random crossover points, then exchange the genes of the chromosome, as shown in Figure 3; if the crossover rate is *x*_*c*_, the number of chromosomes is *y*_*c*_, the crossover quantity is *x*_*c*_ × *y*_*c*_ = *n*_*c*_; a better efficiency could be achieved if crossover rate is 0.75~0.95 [47]. 

**Figure 4:**
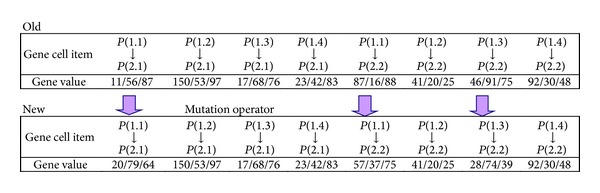
Mutation.

*Step  5*. Randomly select a chromosome for mutation with its position; if the mutation rate is *x*_*m*_, and the number of genes is *y*_*m*_, the mutation number is *x*_*m*_ × *y*_*m*_ = *n*_*m*_, as shown in Figure 4; a higher mutation rate means a higher amplitude, a mutation rate < 0.1 is a typical value [48]. 

*Step  6*. When the chromosomes of best offspring are superior to those of worst population, this population will be replaced as a new one.

*Step  7*. Repeat Steps  2 to 6 until the stop conditions are satisfied.

#### 3.3.2. PSO Solving Model

*Step  1*. Set the particle number, iteration number, maximum speed, learning factor, and inertia weight. 

*Step  2*. Randomly generate the initial speed and position of every particle, with the range specified in the constraint (4)~(12). 

*Step  3*. Substitute the particles of population into the objective function equation (3) to obtain the Pbest of each particle and the Gbest of the population. 

*Step  4*. Update the speed and position of particles using Inertia Weight Method [49]; as shown in (13), when *w* ranges between 0.9 and 1.2, there is a higher opportunity to determine a global optimal solution [50] as follows:

(13) $$\begin{array}{c} v_{i}^{n}=wv_{i}^{c}+c_{1}\times \text{rand}\left(\right)\times \left(s_{i}^{\prime }-s_{i}^{c}\right)+c_{2}\times \text{rand}\left(\right)\times \left(s_{i}^{\prime \prime }-s_{i}^{c}\right), \end{array}$$

(14) $$\begin{array}{c} s_{i}^{n}=s_{i}^{c}+v_{i}^{n}, \end{array}$$

where *v*_*i*_^*c*^ represents the speed when the particle position is changed, *v*_*i*_^*n*^ is the new speed of particle *i*, *s*_*i*_^*c*^ is the current position of particle, *s*_*i*_′ is the memory value of the individual best position of particle *i*, *s*_*i*_′′ is the memory value of the global best position, *s*_*i*_^*n*^ is the new position of particle *i*, *w* is inertia weight, rand() is a random variable between (0,1), and *c*_1_ and *c*_2_ are learning factors. 

*Step  5*. Substitute the updated particle position into the constraint (4)~(12); review if the updated position meets the requirements of constraints; if any particle exceeds the range, update again until all particles meet the requirements of constraint (4)~(12). 

*Step  6*. Compare the particle's objective function value with updated particle position and its Pbest. If the objective function value is superior to the Pbest, the Pbest of this particle will be replaced by its objective function value with updated particle position. 

*Step  7*. Compare the Pbest and Gbest. If the best value of particle is superior to Gbest, the Gbest will be replaced by the best value of the particle. 

*Step  8*. Repeat Steps 2 to 7 until meeting the stop conditions or reaching the set iteration number.

#### 3.3.3. PSO-GA Solving Model

*Steps  1~3*. Perform Steps 1~3 of PSO solving model. 

*Step  4*. Perform Step  3 of GA solving model to select the particle positions of better fitness functions stored in the library. With this selection mechanism, there is a higher probability that the particle position of better fitness functions will become Gbest; another new Gbest could be obtained through recombination, and the particles could be evaluated and properly deleted, of which the worst individuals are replaced by optimum individuals, after sequencing of fitness functions. 

*Step  5*. Obtain a new group of good populations using the selection mechanism, then perform Steps 1~3 of PSO solving model and obtain the new Pbest and Gbest. 

*Step  6*. Perform Step  5 of GA solving model to randomly select mutation particles for single-point mutation and generate a new population. 

*Step  7*. Perform Steps 4~7 of PSO solving model to obtain the new Pbest and Gbest of the new population. 

*Step  8*. Repeat Steps 2 to 7 until stop conditions are met.

#### 3.3.4. PSO-SA Solving Model

*Steps  1~6*. Perform Steps  1~6 of PSO solving model. 

*Step  7*

*Substep  7.1*. Set start temperature (*T*_start_), end temperature (*T*_end_), cooling rate (*α*), and length of Markov Chain (*M*).

*Substep  7.2*. Disturb the updated particle under current temperature *T*, and generate a neighboring solution *k*. 

*Substep  7.3*. Calculate the objective function, perform disturbance mechanism under current temperature to generate a neighboring solution *k*, and then substitute it into the objective function equation (5), and calculate its fitness function *f*(*X*) and the difference of the fitness function Δ*f*(*n*) = *f*(*X*′) − *f*(*X*). Obtain initial solution *X*, and through iteration of random disturbances neighboring reasonable solutions, obtain a new solution *X*′; every particle runs across the length of Markov Chain *M* several times at start temperature *T*_start_; a fitness is obtained every time (energy function Δ*f*(*n*)). 

*Substep  7.4*. Judge if this neighboring solution is accepted through probability function, using probability function (15), as shown below, and then randomly generate random number *r* from 0 to 1 as follows:

(15) $$\begin{array}{c} P\left(n\right)=\{\begin{array}{cc} 1, & \text{if}\begin{array}{c} \;\Delta f\left(n\right)\leq 0, \end{array}\\ \exp\left(\frac{-\Delta f\left(n\right)}{T}\right), & \text{if}\begin{array}{c} \;\Delta f\left(n\right)>0, \end{array} \end{array} \end{array}$$

*Substep  7.5*. Compare random number *r* with probability number *P*(*n*); if *r* ≤ *P*(*n*), it will disturb the neighboring solution *k*; replace the particle and its fitness function; if *r* > *P*(*n*), it will not replace the particle. A new disturbance solution will generate if not accepted, until the termination of set search times.

*Substep  7.6*. Repeat Substeps  7.2–7.5 through Markov Chain (*M*) until *M* times, and then perform the cooling steps, indicating that a stable state is reached under this temperature. 

*Substep  7.7*. Implement the cooling procedure at the set-cooling rate of *α* through a cooling mechanism *T* = *T* × *α*. 

*Substep  7.8*. Judge if the cycling is finished through the set end temperature *T*_end_, if *T* ≤ *T*_end_, perform the next step; if *T* > *T*_end_, repeat Substeps 7.2 to 7.7 until *T* ≤ *T*_end_. 

*Step  8*. Repeat Steps 2 to 7 until reaching the desired iteration number.

#### 3.3.5. GA-SA Solving Model

*Steps  1~5*. Perform Steps 1 to 5 of GA solving model. 

*Step  6*. Perform Substeps 7.1 to 7.8 of PSO-SA solving model. 

*Step  7*. Generate new offspring through genetic evolution; if optimum fitness function of offspring is superior to that of the population will be replaced as a new one otherwise, maintain the chromosomes of original population for next-generation evolution. 

*Step  8*. Repeat Steps 3 to 7 until the set stop conditions are met.

## 4. Illustrative Example and Results Analysis

### 4.1. Case Description

In a complex supply chain network, even a leading manufacturer cannot guarantee 100% yield during the production process, or prevent any defect during the transport process. However, the defect ratio and loss ratio are not fixed; thus, fuzzy defect ratio and fuzzy transport loss ratio are applied in this paper.

**Figure 5:**
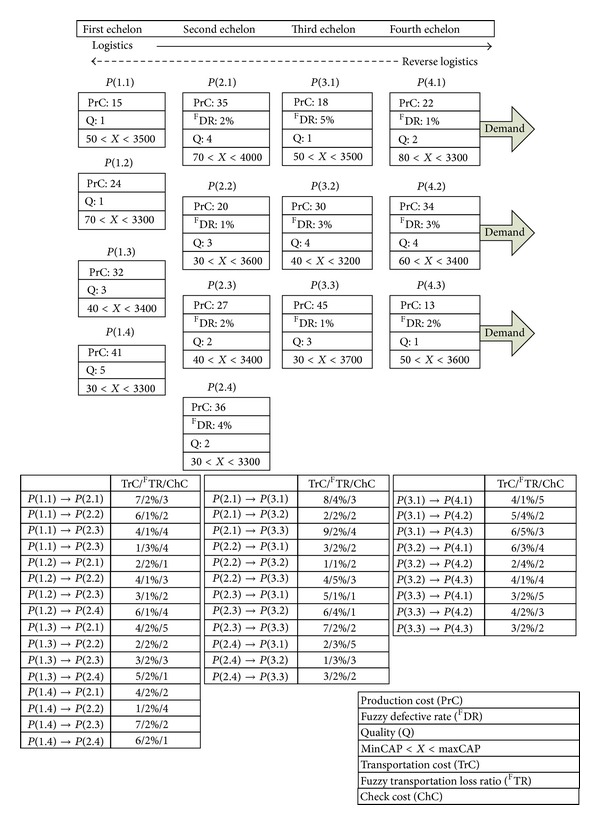
{4-4-3-3} reverse supply chain network.

Based on a supply chain network of {4-4-3-3}, this paper simulated rework activity of returned defective products through a multi-product, multi-phase production plan. Assuming that the initial inventory is zero, the defective products arising from the production process of upstream manufacturers, and from the transport process, are returned to original manufacturers for rework; the suppliers of 1st–4th hierarchy have no fixed production defect ratio or transport loss ratio, meanwhile the multi-product, multi-phase production is planned into three phases, with defective products only returned during the second phase. Moreover, assuming that the production defect ratio and check costs of the first-hierarchy suppliers are not considered, only the defective products from the partners of 2nd to the 4th are returned for rework; in addition, assume that the rework process is the same as the production process, then the rework costs and production costs are the same. According to first-phase production planning, *X* products for final customer requirements amount to 400, 350, 450, and *Y* products amount to 250, 150, 200, and *Z* products amount to 100, 200, and 250.  Figure 5 depicts the framework of a reverse supply chain and relevant parameters, including: production costs (PrC), transport costs (TrC), check costs (ChC), quality (*Q*), fuzzy defect ratio (^F^DR), fuzzy transport loss ratio (^F^TR), and maximum and minimum capacity (Max. CAP. and Min. CAP.). Since the production defect ratio and transport loss ratio are fuzzy sets with triangular fuzzy number, (1) is used for defuzzification. For partner *P*(2.1), its fuzzy number of defect ratio is (0.6%, 2.2%, 3%), then its ^F^DR is 2% according to (1). For the conciseness of this paper, the detail calculating processes of the production defect and transport loss ratios for all partners and transport paths are not presented, and their defuzzified values are shown in Figure 5.

### 4.2. Experimental Results and Analysis

PSO, GA, PSO-GA, GA-SA, and PSO-SA were applied for solving the distribution issues of a reverse supply chain network, the experimental design was conducted under the parameters of solving performance, of which every combination of parameters was implemented 20 times to determine an optimal combination, as listed in Table 1.

It is learnt from Table 1 that the optimal combination of PSO parameters is particle number 10, generation number 1000, maximum speed 1.25, inertia weight 2.15, and learning factor 2.05; the optimal combination of GA parameters is population number 10, generation number 1000, crossover rate 0.8, and mutation rate 0.07; the optimal combination of PSO-GA parameters is particle number 20, generation number 500, maximum speed 1.25, inertia weight 2.15, learning factor 2.05, and mutation rate 0.07; the optimal combination of GA-SA parameters is: population number 10, generation number 500, crossover rate 0.8, mutation rate 0.07, start temperature 300, Markov Chain 50, cooling rate 0.9, and end temperature 1; the optimal combination of PSO-SA parameters is: particle number 10, generation number 1000, maximum speed 1.25, inertia weight 2.15, learning factor 2.05, start temperature 400, Markov Chain 100, cooling rate 0.99, end temperature 5.

To compare the advantages and disadvantages of these algorithms, ANOVA was used to judge if convergence value, execution time, and convergence time differed considerably, then the algorithms were compared using Scheffe's multiple comparison method [51]; one-way ANOVA was used to check the difference of characteristics and variables [52]; Scheffe's multiple comparison method was then used to check the differences of the various groups, and whether the differences reached a significant level. Tables 2, 3, and 4 list the comparative check data of the five algorithms, which were sourced from 30 occasions of independent calculations for the optimal combination of parameters.

In Tables 2–4, the results of ANOVA verification are shown that all H_0_ are rejected (*P* value < *α* = 0.05). Then, the fitness value, execution time, and convergence time of the algorithms were compared using Scheffe's multiple comparison method, with Scheffe's equation as follows:

(16) $$\begin{array}{c} \bar{x_{i}}-\bar{x_{j}}-\sqrt{\left(k-1\right)F_{\alpha (k-1)(n-k)}}\sqrt{\text{MSE}\left(\frac{1}{n_{i}}+\frac{1}{n_{j}}\right)},\\ \bar{x_{i}}-\bar{x_{j}}+\sqrt{\left(k-1\right)F_{\alpha (k-1)(n-k)}}\sqrt{\text{MSE}\left(\frac{1}{n_{i}}+\frac{1}{n_{j}}\right)}, \end{array}$$

where $\bar{x_{i}}$, ${\bar{x}}_{j}$ are the mean values of algorithms compared, *k* is group freedom, *F*_*α*(*k*−1)(*n*−*k*)_ is critical value, MSE is intergroup MS, *n*_*i*_, *n*_*j*_ is number of samples.

Table 5 shows ^Fitness^ *μ*_PSO-GA_ = ^Fitness^ *μ*_GA-SA_ = ^Fitness^ *μ*_PSO-SA_ < ^Fitness^ *μ*_PSO_ = ^Fitness^ *μ*_GA_; that is, PSO-GA, GA-SA, and PSO-SA are all better than PSO and GA, and there are no clear differences in the fitness values of the three algorithms. The comparative result of system execution time is shown in Table 6, and ^ET^ *μ*_PSO_ < ^ET^ *μ*_PSO-GA_ < ^ET^ *μ*_PSO-SA_ < ^ET^ *μ*_GA-SA_ < ^ET^ *μ*_GA_ is the PSO is superior to other solving models. The convergence times of the algorithms are shown in Table 7, and ^CT^ *μ*_PSO-GA_ < ^CT^ *μ*_PSO_ < ^CT^ *μ*_PSO-SA_ < ^CT^ *μ*_GA-SA_ < ^CT^ *μ*_GA_ PSO-GA < PSO < PSO-SA < GA-SA < GA; that is, PSO-GA, has faster convergence speed than PSO, PSO-SA, GA-SA, and GA. The results show that PSO-GA performs better in fitness value, execution time, and convergence time. Then, the PSO-GA is employed to make the distribution plan of three periods reverse supply chain and the results are shown in Tables 8, 9, and 10.

## 5. Conclusions

This paper focused on analyzing the issues of returning defective products to original manufacturers in a reverse supply chain system. Fuzzy defect and fuzzy transport loss ratios were considered, and an optimized mathematical model was developed. This model combined cost and quality with T-technology and described how to plan a reverse supply chain on the precondition of meeting customer demands and realizing the capacity of partners. To solve the problems efficiently, three hybrid algorithms were applied to this reverse model, including PSO-GA, PSO-SA, and GA-SA; then, the performances of these algorithms were compared. The experimental results show that if the fitness value, execution time, and convergence time are considered, PSO-GA has the minimal value, which means that PSO-GA has the qualities and capabilities for dealing with the production planning and distribution of a multi-phase, multi-product reverse supply chain.

## Figures and Tables

**Table 1:** Combination of experimental parameters.

PSO	Population size	10	10	10	10	20	20	20	20
	Generations	500	500	1000	1000	500	500	1000	1000
	Max velocity	0.95	1.25	0.95	1.25	0.95	1.25	0.95	1.25
	Initial weight	1.25	2.15	1.25	2.15	1.25	2.15	1.25	2.15
	*c*_1_, *c*_2_	2.05	2.05	2.05	2.05	2.05	2.05	2.05	2.05
	Avg. fitness	24462289	24461153	24460103	24458116	24459087	24460841	24458923	24457531
	Avg. execution time (sec.)	6.731	6.896	12.468	12.391	12.842	12.546	21.391	21.016
	Avg. convergence time (sec.)	4.766	4.275	8.791	8.437	7.986	8.311	17.694	16.972

GA	Population size	5	5	5	5	10	10	10	10
	Generations	500	500	1000	1000	500	500	1000	1000
	Crossover rate	0.75	0.8	0.75	0.8	0.75	0.8	0.75	0.8
	Mutation rate	0.08	0.07	0.08	0.07	0.08	0.07	0.08	0.07
	Avg. fitness	24465387	24464085	24462752	24461924	24463297	24463159	244603191	24459450
	Avg. execution time (sec.)	19.373	18.859	67.693	68.047	47.375	47.734	225.836	217.512
	Avg. convergence time (sec.)	17.716	16.827	62.549	62.764	44.507	44.642	219.675	211.741

PSO-GA	Population size	10	10	10	10	20	20	20	20
	Generations	500	500	1000	1000	500	500	1000	1000
	Max velocity	0.95	1.25	0.95	1.25	0.95	1.25	0.95	1.25
	Initial weight	1.25	2.15	1.25	2.15	1.25	2.15	1.25	2.15
	*c*_1_, *c*_2_	2.05	2.05	2.05	2.05	2.05	2.05	2.05	2.05
	Mutation rate	0.08	0.07	0.08	0.07	0.08	0.07	0.08	0.07
	Avg. fitness	24790482	24692938	24662117	24579829	24507556	24453060	24697893	24662084
	Avg. execution time (sec.)	6.758	6.579	11.864	12.714	12.898	12.685	23.257	22.934
	Avg. convergence time (sec.)	3.147	3.038	5.342	6.182	6.379	5.824	12.681	12.507

GA-SA	Population size	5	5	5	5	10	10	10	10
	Generations	500	500	1000	1000	500	500	1000	1000
	Crossover rate	0.75	0.8	0.75	0.8	0.75	0.8	0.75	0.8
	Mutation rate	0.08	0.07	0.08	0.07	0.08	0.07	0.08	0.07
	Initial temperature	300	300	400	400	300	300	400	400
	Markov Chain Length	50	50	100	100	50	50	100	100
	Cooling rate	0.9	0.9	0.99	0.99	0.9	0.9	0.99	0.99
	Final temperature	1	1	5	5	1	1	5	5
	Avg. fitness	24459076	24458365	24458351	24458067	24457247	24456391	24457425	24457544
	Avg. execution time (sec.)	27.477	26.041	81.462	79.039	68.671	66.993	279.145	274.338
	Avg. convergence time (sec.)	22.374	20.768	74.744	73.412	63.467	62.522	265.074	263.862

PSO-SA	Population size	10	10	10	10	20	20	20	20
	Generations	500	500	1000	1000	500	500	1000	1000
	Max velocity	0.95	1.25	0.95	1.25	0.95	1.25	0.95	1.25
	Initial weight	1.25	2.15	1.25	2.15	1.25	2.15	1.25	2.15
	*c*_1_, *c*_2_	2.05	2.05	2.05	2.05	2.05	2.05	2.05	2.05
	Initial temperature	300	300	400	400	300	300	400	400
	Markov Chain Length	50	50	100	100	50	50	100	100
	Cooling rate	0.9	0.9	0.99	0.99	0.9	0.9	0.99	0.99
	Final temperature	1	1	5	5	1	1	5	5
	Avg. fitness	24457588	24457887	24455842	24455147	24455895	24456068	24456457	24456778
	Avg. execution time (sec.)	14.073	13.422	26.087	25.971	26.479	25.706	38.926	37.433
	Avg. convergence time (sec.)	6.102	5.674	11.479	10.347	11.887	12.624	19.211	16.708

**Table 2:** ANOVA verification of fitness values.

Hypothesize						
H_0_: ^Fitness^ *μ*_PSO_ = ^Fitness^ *μ*_GA_ = ^Fitness^ *μ*_PSO-GA_ = ^Fitness^ *μ*_GA-SA_ = ^Fitness^ *μ*_PSO-SA_						
H_1_: otherwise						
Algorithm	Numbers		Sum	Average		Variance

PSO	30		734139577	24471319.23		1120394034
GA	30		734406462	24480215.4		910941494
PSO-GA	30		733498321	24449944.03		1372449396
GA-SA	30		733929774	24464325.8		752499584
PSO-SA	30		733666393	24455546.43		3467767352

Source	SS	Freedom	MS	*F*	*P* value	Critical value

Intergroup	1.76*E* + 10	4	4389633912	2.87880644	0.02486	2.434065
Intragroup	2.21*E* + 11	145	1524810372			
Sum	2.39*E* + 11	149				

Result: reject H_0_						

**Table 3:** ANOVA verification of execution time.

Hypothesize						
H_0_: ^ET^ *μ*_PSO_ = ^ET^ *μ*_GA_ = ^ET^ *μ*_PSO-GA_ = ^ET^ *μ*_GA-SA_ = ^ET^ *μ*_PSO-SA_						
H_1_: otherwise						
Algorithm	Numbers		Sum	Average		Variance

PSO	30		372.022	12.40073		0.076341375
GA	30		6524.661	217.4887		0.165604217
PSO-GA	30		380.424	12.6808		0.076438303
GA-SA	30		2004.847	66.82823		0.187643495
PSO-SA	30		775.495	25.84983		0.192059868

Source	SS	Freedom	MS	*F*	*P* value	Critical value

Intergroup	908155.7	4	227038.9	1626150.094	0.00000	2.434065
Intragroup	20.24453	145	0.139617			
Sum	908176	149				

Result: reject H_0_						

**Table 4:** ANOVA verification of convergence time.

Hypothesize						
H_0_: ^CT^ *μ*_PSO_ = ^CT^ *μ*_GA_ = ^CT^ *μ*_PSO-GA_ = ^CT^ *μ*_GA-SA_ = ^CT^ *μ*_PSO-SA_						
H_1_: otherwise						
Algorithm	Numbers		Sum	Average		Variance

PSO	30		246.605	8.220167		0.0913266
GA	30		6375.408	212.5136		0.7256669
PSO-GA	30		171.189	5.7063		0.014925
GA-SA	30		1863.747	62.1249		0.4336888
PSO-SA	30		326.895	10.8965		0.2385541

Source	SS	Freedom	MS	*F*	*P* value	Critical value

Intergroup	939149	4	234787.2	780458.96	0.00000	2.434065
Intragroup	43.62068	145	0.300832			
Sum	1.23*E* + 09	89				

Result: reject H_0_						

**Table 5:** Multiple comparison on fitness value.

	^Fitness^ *μ*_PSO_	^Fitness^ *μ*_GA_	^Fitness^ *μ*_PSO-GA_	^Fitness^ *μ*_GA-SA_
^Fitness^ *μ*_GA_	(−, +)			
^Fitness^ *μ*_PSO-GA_	(+, +)	(+, +)		
^Fitness^ *μ*_GA-SA_	(+, +)	(+, +)	(−, +)	
^Fitness^ *μ*_PSO-SA_	(+, +)	(+, +)	(−, +)	(−, +)

**Table 6:** Multiple comparison on execution time.

	^ET^ *μ*_PSO_	^ET^ *μ*_GA_	^ET^ *μ*_PSO-GA_	^ET^ *μ*_GA-SA_
^ET^ *μ*_GA_	(−, −)			
^ET^ *μ*_PSO-GA_	(−, −)	(+, +)		
^ET^ *μ*_GA-SA_	(−, −)	(+, +)	(−, −)	
^ET^ *μ*_PSO-SA_	(−, −)	(+, +)	(−, −)	(+, +)

**Table 7:** Multiple comparison on convergence time.

	^CT^ *μ*_PSO_	^CT^ *μ*_GA_	^CT^ *μ*_PSO-GA_	^CT^ *μ*_GA-SA_
^CT^ *μ*_GA_	(−, −)			
^CT^ *μ*_PSO-GA_	(+, +)	(+, +)		
^CT^ *μ*_GA-SA_	(−, −)	(+, +)	(−, −)	
^CT^ *μ*_PSO-SA_	(−, −)	(+, +)	(−, −)	(+, +)

**Table 8:** Distribution plan by PSO-GA (first period).

From	To	Echelon 2				Echelon 3			Echelon 4		
		*P*(2.1)	*P*(2.2)	*P*(2.3)	*P*(2.4)	*P*(3.1)	*P*(3.2)	*P*(3.3)	*P*(4.1)	*P*(4.2)	*P*(4.3)
Echelon 1	*P*(1.1)	10^a^, 6^b^, 49^c^	73, 3, 6	42, 110, 59	24, 253, 5						
	*P*(1.2)	142, 47, 4	174, 26, 33	34, 18, 2	102, 10, 234						
	*P*(1.3)	157, 24, 57	256, 38, 1	7, 29, 56	22, 11, 55						
	*P*(1.4)	74, 39, 6	76, 17, 21	31, 8, 13	74, 14, 1						

Echelon 2	*P*(2.1)					177, 3, 83	116, 8, 0	82, 103, 25			
	*P*(2.2)					458, 2, 37	109, 14, 17	8, 67, 6			
	*P*(2.3)					53, 100, 60	41, 3, 3	17, 58, 65			
	*P*(2.4)					111, 236, 9	10, 29, 244	92, 11, 30			

Echelon 3	*P*(3.1)								170, 0, 0	100, 0, 174	91, 0, 255
	*P*(3.2)								95, 0, 0	47, 0, 0	123, 0, 0
	*P*(3.3)								139, 253, 101	213, 155, 32	245, 204, 0

^a^Product X; ^b^product Y; ^c^product Z.

**Table 9:** Distribution plan by PSO-GA (second period).

From	To	Echelon 1				Echelon 2				Echelon 3			Echelon 4		
		*P*(1.1)	*P*(1.2)	*P*(1.3)	*P*(1.4)	*P*(2.1)	*P*(2.2)	*P*(2.3)	*P*(2.4)	*P*(3.1)	*P*(3.2)	*P*(3.3)	*P*(4.1)	*P*(4.2)	*P*(4.3)
Echelon 1	*P*(1.1)					100, 76, 12	69, 48, 80	61, 349, 47	18, 55, 0						
	*P*(1.2)					34, 19, 22	0, 27, 0	214, 50, 47	91, 10, 14						
	*P*(1.3)					95, 334, 95	131, 13, 51	8, 17, 40	28, 5, 126						
	*P*(1.4)					111, 11, 0	12, 15, 100	9, 91, 0	125, 2, 15						

		**Reverse**													
Echelon 2	*P*(2.1)	**0, 0, 0**	**3, 0, 0**	**3, 0, 0**	**1, 2, 2**					130, 320, 67	189, 27, 59	10, 85, 0			
	*P*(2.2)	**2, 0, 0**	**4, 0, 0**	**6, 0, 0**	**2, 2, 1**					155, 37, 213	52, 38, 17	0, 29, 0			
	*P*(2.3)	**1, 0, 0**	**1, 0, 0**	**0, 0, 0**	**1, 2, 2**					82, 344, 12	123, 62, 108	81, 94, 10			
	*P*(2.4)	**0, 0, 0**	**1, 0, 0**	**0, 0, 0**	**1, 8, 3**					55, 0, 51	139, 7, 0	55, 63, 90			

						**Reverse**									
Echelon 3	*P*(3.1)					**6, 0, 0**	**15, 0, 0**	**2, 0, 0**	**4, 13, 7**				41, 303, 0	134, 361, 0	117, 0, 0
	*P*(3.2)					**3, 0, 0**	**3, 0, 0**	**1, 0, 0**	**0, 1, 3**				130, 0, 0	121, 0, 0	60, 396, 0
	*P*(3.3)					**2, 0, 0**	**0, 0, 0**	**0, 0, 0**	**2, 6, 3**				226, 0, 151	143, 0, 304	64, 6, 150

										**Reverse**					
Echelon 4	*P*(4.1)									**3, 0, 0**	**2, 0, 0**	**2, 3, 1**			
	*P*(4.2)									**4, 0, 0**	**2, 0, 0**	**8, 8, 5**			
	*P*(4.3)									**3, 0, 0**	**4, 0, 0**	**7, 6, 3**			

Demand													400, 300, 150	400, 350, 300	250, 400, 150

**Table 10:** Distribution plan by PSO-GA (third period).

From	To	Echelon 1				Echelon 2				Echelon 3			Echelon 4		
		*P*(1.1)	*P*(1.2)	*P*(1.3)	*P*(1.4)	*P*(2.1)	*P*(2.2)	*P*(2.3)	*P*(2.4)	*P*(3.1)	*P*(3.2)	*P*(3.3)	*P*(4.1)	*P*(4.2)	*P*(4.3)
Echelon 1	*P*(1.1)					86, 18, 4	406, 15, 108	30, 12, 53	24, 83, 13						
	*P*(1.2)					34, 60, 36	137, 6, 28	165, 137, 6	35, 159, 30						
	*P*(1.3)					2, 7, 24	30, 1, 217	47, 116, 0	278, 28, 5						
	*P*(1.4)					56, 0, 10	7, 8, 31	86, 102, 22	17, 46, 98						

		**Reverse**													
Echelon 2	*P*(2.1)	**2, 0, 0**	**1, 0, 0**	**2, 0, 0**	**2, 9, 3**					26, 9, 15	33, 23, 49	113, 55, 11			
	*P*(2.2)	**2, 0, 0**	**0, 0, 0**	**4, 0, 0**	**0, 1, 4**					430, 3, 62	37, 27, 318	105, 0, 4			
	*P*(2.3)	**1, 0, 0**	**3, 0, 0**	**0, 0, 0**	**0, 9, 2**					217, 282, 38	8, 22, 38	93, 64, 6			
	*P*(2.4)	**0, 0, 0**	**1, 0, 0**	**0, 0, 0**	**2, 2, 3**					216, 0, 61	22, 183, 48	99, 104, 12			

						**Reverse**									
Echelon 3	*P*(3.1)					**5, 0, 0**	**6, 0, 0**	**3, 0, 0**	**2, 27, 12**				190, 0, 354	213, 0, 0	58, 0, 193
	*P*(3.2)					**5, 0, 0**	**1, 0, 0**	**3, 0, 0**	**3, 5, 7**				93, 253, 0	165, 0, 0	113, 0, 0
	*P*(3.3)					**0, 0, 0**	**0, 0, 0**	**2, 0, 0**	**2, 7, 2**				166, 0, 0	123, 302, 87	230, 24, 6

										**Reverse**					
Echelon 4	*P*(4.1)									**1, 0, 0**	**2, 0, 0**	**3, 6, 2**			
	*P*(4.2)									**5, 0, 0**	**4, 0, 0**	**5, 7, 16**			
	*P*(4.3)									**3, 0, 0**	**2, 0, 0**	**2, 17, 5**			

Demand													450, 250, 350	500, 300, 100	400, 200, 200

## Symbols of Mathematical Model

*n*: Number of products, *n* = 1,2, 3,…, *N*
*N*: Total number
*i*: Hierarchy of supply chain, *i* = 1,2, 3,…, *I*
*I*: Total number of hierarchy of supply chain
*p*: Production phase, *t* = 1,2, 3,…, *T*
*P*: Total production phase
*J*_*i*_: Serial number of partner of *i*th hierarchy
*K*_*i*_: Serial number of partner of *i*th hierarchy designated to original manufacturer
ChC_(*i*,*j*)_: Check cost of *j*th partner of *i*th hierarchy
PrC_(*i*,*j*)_: Production cost of *j*th partner of *i*th hierarchy
TrC_((*i*,*j*),(*i*+1,*k*))_: Transport cost from *j*th partner of *i*th hierarchy to original (designated) partner *k* of *i* + 1 hierarchy
^F^TR_((*i*,*j*),(*i*+1,*k*))_: Fuzzy transport loss ratio from *j*th partner of *i*th hierarchy to original (designated) partner of *i* + 1 hierarchy
^F^DR_(*i*,*j*)_: Fuzzy defect ratio of *j*th partner of *i*th hierarchy
*X*_(*i*,*j*)_^*p*^: Production quantity of *j*th partner of *i*th hierarchy at phase *p*
*X*__((*i*,*j*),(*i*+1,*k*))__^*P*^: Transport quantity from *j*th partner of *i*th hierarchy at phase *p* to original (designated) partner of *i* + 1 hierarchy
*RX*__((*i*+1,*k*)(*i*,*j*))__^*P*−1^: Quantity of defective products transported from *j*th partner of *i*th hierarchy at phase *p* − 1 back to original (designated) partner of *i* + 1 hierarchy
*Max*⁡CAP_(*i*,*j*)_: Maximum capacity of *j*th partner of *i*th hierarchy
*Min*⁡CAP_(*i*,*j*)_: Minimum capacity of *j*th partner of *i*th hierarchy
*Q*_(*i*,*j*)_^*p*^: Quality level of *j*th partner of *i*th hierarchy at phase *p*
ChC_(*i*,*j*)_: Check cost of *j*th partner of *i*th hierarchy
PrC_(*i*,*j*)_: Production cost of *j*th partner of *i*th hierarchy
*Tr*⁡C_((*i*,*j*),(*i*+1,*k*))_: Transport cost from *j*th partner of *i*th hierarchy to original (designated) partner *k* of *i* + 1 hierarchy
SD_(*i*,*j*)_^*Q*^: Standard deviation of quality of *j*th partner of *i*th hierarchy
SD_(*i*,*j*)_^ChC^: Standard deviation of check cost of *j*th partner of *i*th hierarchy
SD_(*i*,*j*)_^PrC^: Standard deviation of production cost of *j*th partner of *i*th hierarchy
SD_((*i*,*j*),(*i*+1,*k*))_^TrC^: Standard deviation of transport cost from *j*th partner of *i*th hierarchy to original (designated) partner *k* of *i* + 1 hierarchy
INT{}: Integer.
